# Serum Concentration of IL-5 Receptor (IL-5R) and Associations with Disease Severity in Patients with Chronic Spontaneous Urticaria (CSU) and Atopic Dermatitis (AD)

**DOI:** 10.3390/ijms25147598

**Published:** 2024-07-11

**Authors:** Krzysztof Gomułka, Maciej Tota, Julia Laska, Karina Gojny, Łukasz Sędek

**Affiliations:** 1 Department of Internal Medicine, Pneumology and Allergology, Wroclaw Medical University, 50-368 Wroclaw, Poland; 2 Student Research Group of Internal Medicine and Allergology, Wroclaw Medical University, 50-368 Wroclaw, Poland; 3 Student Research Group of Microbiology and Immunology, Department of Microbiology and Immunology, Zabrze, Medical University of Silesia in Katowice, 40-055 Katowice, Poland; 4 Department of Microbiology and Immunology, Zabrze, Medical University of Silesia in Katowice, 40-055 Katowice, Poland

**Keywords:** atopic dermatitis, chronic spontaneous urticaria, interleukin-5, interleukin-5 receptor, SCORAD index, UAS7 scale, VAS

## Abstract

The immunological pathogenesis of atopic dermatitis (AD) and chronic spontaneous urticaria (CSU) has not been fully elucidated yet. The aim of our research was to assess the serum concentration of interleukin-5 receptor (IL-5R) in relation to the disease activity and pruritus intensity in adult patients with AD and CSU. This pilot study included 45 participants (15 patients with AD, 15 patients with CSU, and 15 healthy controls). Blood samples were taken to examine the serum levels of IL-5R using the enzyme-linked immunosorbent assay (ELISA) test. The Scoring Atopic Dermatitis (SCORAD) index, the Urticaria Activity Score (UAS7), and the Visual Analogue Scale (VAS) were used to assess the disease activity and the pruritus intensity, respectively. Obtained results revealed that the IL-5R concentration was significantly higher in patients with CSU than in patients with AD and in the controls (*p* = 0.038). There was a positive correlation between the IL-5R level and the SCORAD index in patients with AD (r = −0.9, *p* = 0.047), which was not found for the CSU activity by UAS7 and with the pruritus severity by VAS in both examined groups of patients. Our findings underscore higher serum levels of IL-5R among CSU and AD patients, which may highlight its functional role in the pathogenesis of these diseases. In contrast, IL-5R might not be fully useful in reflecting the severity of symptoms. Although our results are promising, this study should be conducted on a larger cohort of patients.

## 1. Introduction

Chronic spontaneous urticaria (CSU) and atopic dermatitis (AD) are two prevalent dermatological conditions that significantly impact the quality of life of affected individuals [1,2]. These chronic inflammatory skin disorders not only present considerable challenges in diagnosis and management but also necessitate a deeper understanding of their underlying pathophysiology for effective treatment strategies.

**Figure 1:**
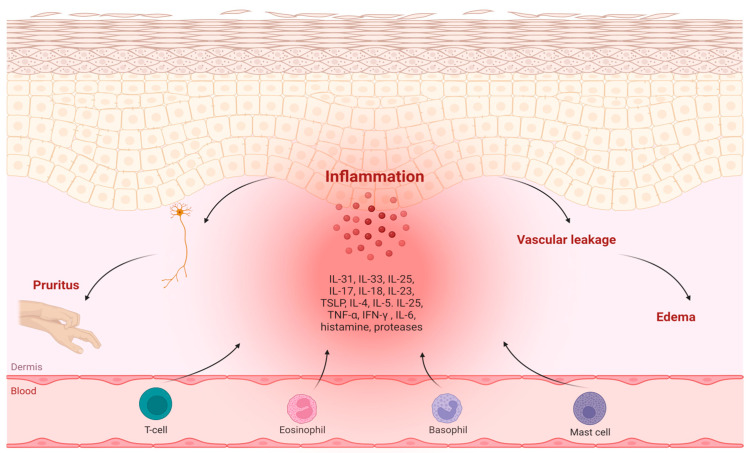
Pathogenesis pathways in chronic spontaneous urticaria.

Urticaria is categorized based on its duration as either acute (less than 6 weeks) or chronic (at least 6 weeks), and its potential to have a triggering factor is either inducible or spontaneous. CSU affects approximately 0.02–2.7% of the population with no evident distinction between children and adults. However, it has a slightly elevated occurrence among patients in fifth and sixth decade of life [3,4,5,6]. In the pathogenesis of CSU, the activation and degranulation of mast cells in the skin trigger the release of histamine and other mediators. This cascade leads to sensory nerve activation (itch), vasodilation (erythema), plasma extravasation (edema), and cellular recruitment, manifesting with such symptoms as itchy wheals (hives) and angioedema [7]. Additionally, beyond the release of histamine from dermal mast cells, nonlesional skin in CSU patients exhibits the upregulation of adhesion molecules, infiltration of eosinophils, altered cytokine expression [8], and sometimes a mild to moderate increase in mast cell numbers [9] (Figure 1).

AD, the most common chronic inflammatory skin disease, affects up to 20% of children worldwide. It persists in adulthood in a minority of cases, affecting up to 3% of the adult population [10,11]. There has been an increased prevalence of AD over the past decades, not only in developed countries but also in developing ones [11]. The diagnosis of AD is primarily based on clinical presentation and patient history, excluding erythematous and eczematous conditions. While diagnosing AD is generally straightforward in infants and young children, it can become particularly challenging in severe cases and adult populations [12]. In infants, skin lesions typically manifest between 2 and 6 months of age. These lesions may present as papules and papulovesicles, which can coalesce into large plaques that exude fluid and form crusts [13]. Although some infants may initially present with flexural disease, which affects areas such as the antecubital and popliteal fossae, wrists, and ankles, this form of the disease typically emerges after the first year of life. In contrast, adult-onset AD exhibits a more heterogeneous presentation, characterized by greater variability in lesion morphology and distribution, with a notable predilection for the head, neck, hands, and feet. Persistent itching leads to scratching and further contributes to skin thickening and lichenification over time [11,14].

**Figure 2:**
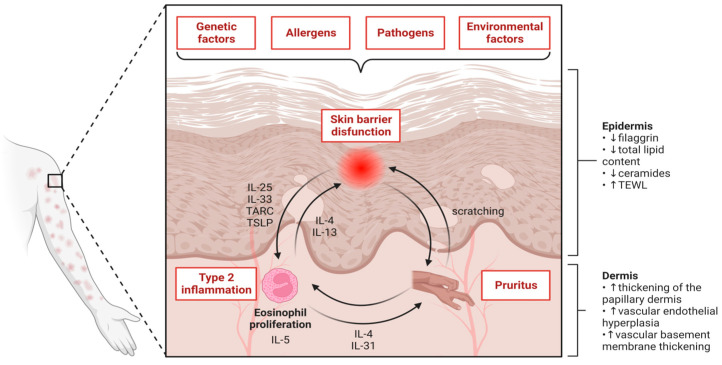
Pathogenesis pathways in atopic dermatitis; TEWL, transepidermal water loss; TARC, thymus and activation-regulated chemokine; TSLP, thymic stromal-derived lymphopoietin.

The epidermis, crucial as a physical and functional barrier, exhibits significant defects in AD skin. Compromised skin barrier function increases permeability and water loss, making the skin more vulnerable to allergens and pollutants. Immune cells like dendritic cells (DCs) and type 2 innate lymphoid cells (ILC2s) are activated by alarmins released by damaged skin cells, such as interleukins (IL)-33, IL-25, and thymic stromal lymphopoietin (TSLP). These activated immune cells produce cytokines such as IL-5 and IL-13, further activating eosinophils and Th2 cells, exacerbating inflammation [10,14]. Nonlesional skin in AD patients exhibits an overt increase in the proportion of Th2 cells [15] (Figure 2).

As mentioned, the pathogenesis of AD and CSU remains incompletely understood, with various mechanisms involved. In the immune response, there is a particular emphasis on the role of the Th2 pathway. Th2 cells are characterized by the production of cytokines, including IL-4, IL-5, IL-6, IL-9, IL-10, and IL-13 along with ILC2 cells, which also produce IL-4, IL-5, and IL-13. These Th2 and ILC2 cells share many functional similarities and are key factors in the development of AD and contribute to the chronicity of CSU [3,10,16,17,18].

**Figure 3:**
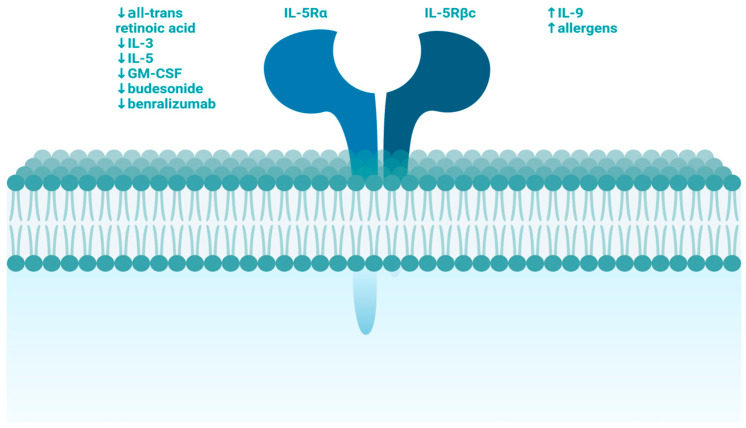
Molecules that upregulate IL-5R expression are presented on the right; molecules that downregulate IL-5R expression are presented on the left.

In our study, we aim to investigate the concentration of IL-5 receptor (IL-5R) within the context of CSU and AD. IL-5 is a well-known pro-inflammatory cytokine secreted by various cell types, such as Th2 lymphocytes, ILC2s, mast cells, and eosinophils. IL-5 influences target cells through the IL-5R, and its functions include promoting eosinophil proliferation, activation, viability, and migration out of the bloodstream towards the site of inflammation [19]. Additionally, IL-5 can increase the expression of IL-2Rα on B cells, boost IgA production, and promote the proliferation and differentiation of both B cells and cytotoxic T cells. IL-5 does not directly participate in IgE production but can stimulate mast cells to release histamine [20,21,22]. Several factors that downregulate and upregulate IL-5R expression are presented in Figure 3.

**Figure 4:**
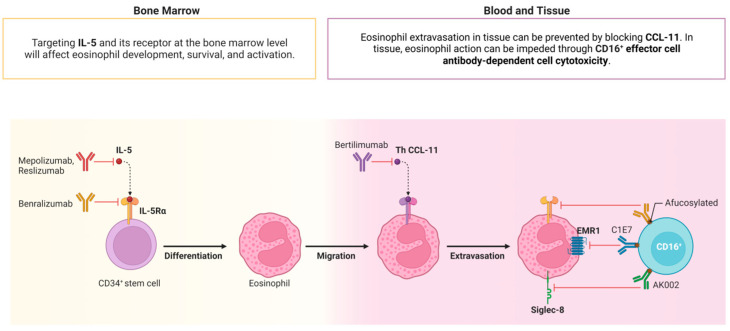
IL-5 and IL5-R targets.

Over the past decade, therapeutic approaches for atopic dermatitis and chronic spontaneous urticaria have mostly focused on alleviating symptoms and decreasing inflammation rather than addressing the underlying cause. However, the emergence of novel biological agents targeting IL-5 and IL-5R, such as mepolizumab, reslizumab, and benralizumab, has introduced new therapeutic options [23,24,25] (Figure 4).

## 2. Results

The obtained results show that the mean IL-5R serum concentration was significantly higher in patients with CSU than in the control group (*p* = 0.038). The values and differences in serum concentrations of IL-5R in patients and the control group are shown in Table 1.

In the AD group, 3/15 (20%) of participants had severe AD, and 12/15 (80%) had a moderate degree of disease severity according to the SCORAD index. Furthermore, we found relationships between the mean IL-5R serum concentration and the severity of AD: there was a significant positive correlation between IL-5R level and the SCORAD index (*p* = 0.047). On the contrary, no significant correlation was found between the IL-5R concentration and the activity of CSU measured with the UAS7 scale. All patients with CSU and AD reported skin pruritus of average to moderate intensity, which was assessed with the use of the VAS. Nevertheless, the mean serum IL-5R concentration did not correlate with the intensity of itching sensations in the examined patients. The data on the disease severity in patients with CSU and AD are shown in Table 2.

## 3. Discussion

The involvement of eosinophils in the inflammatory infiltrate associated with AD has been well documented. It has been observed that both the number of eosinophils and the levels of eosinophil granule proteins in the peripheral blood are elevated in the majority of patients with AD, and these parameters seem to correlate with the activity of the disease [26,27]. Whenever systemic eosinophilic inflammation is identified as a clinically pertinent observation, it is reasonable to consider that inhibiting IL-5 or its receptor might represent a putative therapeutic approach. Mepolizumab, reslizumab, and benralizumab constitute humanized monoclonal antibodies that inhibit IL-5 signalling. While mepolizumab and reslizumab bind directly to IL-5, benralizumab targets the IL-5 receptor alpha (IL-5Rα), subsequently inducing antibody-dependent cell-mediated cytotoxicity (Figure 4) [28]. Clinical trials indicate that reslizumab treatment does not elicit meaningful responses in patients lacking elevated eosinophil levels, implying that the effectiveness of IL-5 inhibition predominantly results from the reduction in eosinophil counts [29].

In a randomized, double-blind, placebo-controlled, parallel-group study, 18 patients diagnosed with AD were administered two single intravenous doses of 750 mg of mepolizumab. In comparison, 22 patients received a placebo treatment. The results indicated a significant reduction in peripheral blood eosinophil counts among the treatment group compared to the placebo group. However, the treatment did not achieve clinical success as determined by the physician’s global assessment, the objective SCORAD, itch scoring metrics, and the atopy patch test (APT) [30,31]. The findings from recent research indicate that anti-IL-5 therapies might not yield efficacy in treating AD due to their failure to decrease eosinophil levels in the skin substantially. Alternatively, it is hypothesized that eosinophils may not play a critical role in the pathogenesis of AD. To further explore these possibilities, forthcoming studies should consider administering increased doses of anti-IL-5 and subsequently assess the infiltration of eosinophils into lesional skin areas [32].

Elevated levels of serum-soluble IL-5 α (s-IL-5Rα) are observed prior to the onset of AD in pediatric populations. A substantial correlation was identified between eczema phenotypes and s-IL-5Rα. Following adjustments for multiple comparisons, it was observed that children with late-onset AD exhibited significantly elevated levels of s-IL-5Rα in comparison to individuals who had never experienced AD [33]. In a study by Taha et al., acute and chronic skin lesions demonstrated a marked elevation in the quantity of IL-5Rα and GM-CSFRα mRNA-positive cells compared to uninvolved AD skin and normal skin. Chronic skin lesions exhibited a significantly higher prevalence of IL-5Rα and GM-CSFRα mRNA-positive cells in comparison to acute AD skin [34]. It has been observed that the rs2522411 SNP and the T-A haplotype in the *IL-5* gene, along with serum IL-5 levels, exhibit a strong association with the extrinsic (allergic) type of AD, as opposed to the intrinsic (nonallergic) type of AD. IL-5 is crucial in the proliferation and survival of eosinophils, and thus, IL-5 plays an important role in allergic type of AD. Furthermore, the correlation between the rs6771148 SNP and the T-C-T haplotype in the *IL-5Rα* gene with blood eosinophil counts and serum ECP (eosinophil cationic protein) levels suggests a role for the *IL-5Rα* gene in the regulation of eosinophils in the peripheral blood [35].

CSU is predominantly regarded as a disease driven by mast cell activation. Emerging evidence suggests that eosinophils may also play a role in the manifestation of symptoms. In a study by Di Lorenzo et al., an increase in both blood eosinophil counts and serum ECP levels was found in patients presenting with acute urticaria [36]. Another study showed increased EDN (eosinophil-derived neurotoxin) levels in patients with CSU [37]. As EDN is secreted predominantly by eosinophils, this biomarker may serve as a direct indicator of exacerbations in eosinophilic inflammation [38]. Although numerous allergic and inflammatory conditions are frequently correlated with an increase in peripheral blood eosinophils, the situation may be reversed in CSU, where a reduction in peripheral blood eosinophils, or eosinopenia, is often observed in many patients. Potential mechanisms for this phenomenon may involve the depletion of blood eosinophils due to their recruitment into the skin during active phases of the disease, and their immunological destruction within the bloodstream. The incidence of eosinopenia was significantly elevated in patients with CSU compared to that observed in the general population. Eosinopenia was detected in approximately 10% of patients with CSU compared to 5% of the general population. Eosinopenia in patients with CSU is associated with elevated disease activity, type IIb autoimmunity, and suboptimal response to therapeutic interventions [39].

A 24-week repeated-measures study was conducted to evaluate the clinical efficacy of benralizumab in CSU. The values of UAS7 and CUQoLTS (Chronic Urticaria Quality-of-Life Questionnaire Total Score) were significantly correlated after administration compared to the baseline. Significant improvement was observed in the CUQoLTS components, specifically in the pruritus/wheal scores and the urticarial interference with physical activities, sleep, and leisure time. The findings of this study advocate for the administration of benralizumab in treating unresponsive CSU to standard glucocorticoid-antihistamine therapy (SGAH). The improvements related to benralizumab in UAS7 and CUQoLTS are comparable to those reported for omalizumab in the treatment of CSU, as indicated in prior research [40,41]

The role of IL-5 in AD and CSU is well documented, with numerous lines of evidence supporting its significant contribution to the pathogenesis of these diseases. In our study, there was a significant association between IL-5R serum concentrations in patients with CSU compared to healthy individuals, suggesting that anti-IL-5 therapies may be successful in that group. In summary, the presented pilot study is one of the reports to assess an increased serum level of IL-5R in adult patients with CSU and AD compared to a healthy control group. Moreover, the IL-5R level was found to be correlated to AD severity measured by the SCORAD index. In contrast, this molecule was irrelevant to CSU activity by UAS7 and itch intensity by VAS in examined patients. Therefore, our results support the concept of a systemic and inflammatory component in CSU and AD and might be useful in the investigations of the complex, multi-pronged, and intertwined etiology of these diseases. Nevertheless, further studies on a larger cohort of patients should be conducted to clarify whether IL-5R may be a potential biomarker of CSU and AD, prognostic tool prior to exacerbations, or new effective targeted therapies in the era of personalized medicine. We suggest that future clinical trials should take into account markers, such as peripheral blood eosinophil counts and QoL questionnaires assessed before and after the treatment.

The current study has several limitations. The primary limitation of our pilot study is the small patient group that was examined. Thus, for additional validation, we believe a more extensive examination ought to be carried out. Moreover, the current study employed a cross-sectional design, which provides data from only one point in time, possibly neglecting changes over time or causal connections that might develop throughout the prolonged course of the disease.

## 4. Materials and Methods

### 4.1. Examined Groups

This pilot study included 45 participants (35 females and 10 males) aged 20 to 56 (average age: 30.91 ± 8.65 years). The study group was composed of two subgroups: 15 subjects diagnosed with CSU, based on the medical history and complex physical examination before participation in our study, and 15 subjects diagnosed with AD according to criteria defined by Hanifin and Rajka [42]. The control group consisted of 15 healthy volunteers with a negative medical history of chronic skin diseases. The demographic data are shown in Table 3.

The exclusion criteria for all subjects were as follows: lack of consent, age under 18 years or over 70 years, treatment with long-acting antihistamines or systemic corticosteroids (up to 14 days prior to the study), females during pregnancy, significant comorbidities, and an unstable period of the skin disease. The study protocol was approved by the local ethical committee (Bioethics Committee at Wroclaw Medical University, Wroclaw, Poland; protocol code KB–224/2020), and all participants signed informed consent.

### 4.2. Blood Collection and Biochemical Analysis

Peripheral blood samples (5 mL) were collected to the coagulation activator tubes (Saerstedt AG & Co., Nümbrecht, Germany) from all patients with CSU, AD, and the healthy controls. Then, the serum was separated after centrifugation at room temperature at 1700 rpm for 16 min. and kept at a temperature of −70 °C in an Eppendorf tube (AG, Hamburg, Germany) until further analysis. Single measurements of the serum concentrations of IL-5R were performed with the human enzyme-linked immunosorbent assay (ELISA) kits, following manufacturers’ instructions (Wuhan EIAAB Science Co., Ltd., Darmstadt, Germany).

### 4.3. Disease Severity

The Urticaria Activity Score (UAS) was used to assess disease activity in patients with urticaria. The questionnaire analyses the number of wheals and the intensity of pruritus in a recommended 7-day monitoring period (UAS7), with once-daily documentation (resulting in a summary score from 0 to 42 points) in patients with CSU [7]. The Scoring Atopic Dermatitis (SCORAD) index was used to assess the severity of AD. Based on the estimated score, patients can be subdivided into mild (<25 points), moderate (25–50 points), or severe (>50 points) AD [43]. Additionally, the Visual Analogue Scale (VAS) was applied to evaluate the difference in intensity of pruritus observed in subsequent weeks. Patients with CSU and AD were asked to assess the intensity of the itch, from 0 points (no itching) to 10 points (worst imaginable itch). The results were ranked as follows: 0–2.9 points—mild pruritus; 3–6.9 points—moderate pruritus; 7–8.9 points—severe pruritus; and 9–10 points—very severe pruritus [44].

### 4.4. Statistical Analysis

Statistical analysis was performed using the Statistica 13.3 software package (StatSoft, Kraków, Poland). To determine the significance of the results, the Bartlett test, the U Mann–Whitney test, the χ^2^ test, and the Spearman correlation coefficient test were used. A *p*-value less than 0.05 was considered statistically significant.

## Figures and Tables

**Table 1:** Laboratory findings in examined participants.

IL-5R	CSU Group (*n*= 15)	AD Group (*n* = 15)	Control Group (*n* = 15)
Mean ± SD (pg/mL)	37.92 ± 4.68	35.83 ± 3.79	27.12 ± 3.23
Minimal–Maximal Value (pg/mL)	31.55–42.03	23.21–74.57	24.82–51.25
*p*-value			
CSU Group vs. Control Group	0.038		
AD Group vs. Control Group	0.151		
CSU Group vs. AD Group	0.348		

IL-5R—interleukin-5 receptor; CSU—chronic spontaneous urticaria; AD—atopic dermatitis; SD—standard deviation; vs.—versus.

**Table 2:** Clinical characteristics of the examined patients with CSU and AD.

Parameters Mean ± SD (Points) Minimal–Maximal Value (Points)	CSU Group	AD Group	Correlation with IL-5R
UAS7 scale	31.0 ± 4.95 22–37	-	r = −0.3; *p* = 0.847
SCORAD index	-	40.6 ± 17.20 26–70	r = −0.9; *p* = 0.047
Pruritus (VAS)	6.4 ± 1.67 4–8	6.0 ± 2.12 4–9	CSU group: r = 0.2; *p* = 0.746 AD group: r = 0.1; *p* = 0.785

UAS, Urticaria Activity Score; SCORAD, Scoring Atopic Dermatitis; VAS, Visual Analogue Scale; IL-5R, interleukin-5 receptor; CSU, chronic spontaneous urticaria; AD, atopic dermatitis; SD, standard deviation.

**Table 3:** Demographic data of the examined groups.

Parameters	CSU Group	AD Group	Control Group
Participants, n	15	15	15
Age (years), mean ± SD	43.2 ± 10.62	30 ± 11.31	31.67 ± 6.77
Sex female, n (%)	12 (80%)	12 (80%)	11 (73.33%)
BMI (kg/m^2^), mean ± SD	29.2 ± 7.9	27.8 ± 6.3	26.7 ± 6.8
Smoker—current or former, n (%)	7 (46.67%)	8 (53.33%)	6 (40%)
Other concomitant atopic disease: (asthma, rhinitis), n (%)	3 (20%)	5 (33.33%)	3 (20%)

AD—atopic dermatitis; BMI—body mass index; CSU—chronic spontaneous urticaria; SD—standard deviation.

## Data Availability

Data sharing is not applicable as no datasets were generated or analysed during the current study.
